# Evaluation of the Psychometric Properties of the Health Assessment Questionnaire (HAQ) in a Population of Individuals With Multiple Sclerosis

**DOI:** 10.3389/fneur.2022.847786

**Published:** 2022-03-28

**Authors:** Anna Berardi, Antonella Conte, Lucilla Cimmino, Carlotta Cimmino, Viola Baione, Sebastiano Giuseppe Crisafulli, Marco Tofani, Matteo Tartaglia, Giovanni Fabbrini, Giovanni Galeoto

**Affiliations:** ^1^Department of Human Neurosciences, Sapienza University of Rome, Rome, Italy; ^2^IRCSS Neuromed, Pozzilli, Italy; ^3^Sapienza University of Rome, Rome, Italy; ^4^Department of Public Health and Infectious Disease, Sapienza University of Rome, Rome, Italy

**Keywords:** disability, occupational therapy, rehabilitation, psychometric properties, multiple sclerosis

## Abstract

**Introduction:**

The Health Assessment Questionnaire (HAQ) has been translated into many languages and it has been classified as the predictor of disability and medical costs, however, the psychometric properties of the HAQ have never been studied in a population with neurological disease. The purpose of this study was the evaluation of the psychometric properties of HAQ in a population of individuals with multiple sclerosis (MS).

**Materials and Methods:**

This cross-sectional study was conducted with patients diagnosed with MS. The evaluation tools administered were the 36-item short form health survey (SF–36) to evaluate the health state of the patients and HAQ and to evaluate the limitations of the activities of daily living (ADL).

**Results:**

A total of 34 patients were included in this study. Cronbach's alpha assessed the internal consistency of the HAQ, and it is equal to 0.94. The study revealed some significant correlations between the dimensions of the SF-36 and the sub-categories of the HAQ using Pearson's Correlation Coefficient. Significant correlations emerged between the demographic and clinical characteristics of patients and the subcategories of HAQ.

**Discussion:**

The HAQ is a valid and reliable tool to assess the limitations of the activities of daily living, and it could provide for the healthcare and rehabilitation sector with an additional evaluation tool.

## Introduction

Chronic symptoms of multiple sclerosis (MS), such as physical functioning impairment, cognitive impairment, emotional burden, and fatigue, considerably affect the quality of life (QOL) of people with MS with an important impact on daily activities and social participation. More than half of the individuals with MS reported physical symptoms that negatively affected activities of daily living (ADLs), such as weakness, problems with balance/coordination, heat/cold sensitivity, numbness/tingling, and trouble moving/muscle stiffness; most patients also reported fatigue and low energy. People also reported a negative effect on emotional and social factors, including self-esteem, general outlook, wellbeing, maintaining/starting relationships, ability to advance in one's career/keep one's job, and coping with life roles (1).

Thus, these symptoms have an important impact on the ability to participate in meaningful activities with repercussions on individuals and societies (2–4). Overall, participation is defined as the “involvement in a life situation,” which includes daily activities, leisure, social activities, and work (5, 6). Participation is associated with life quality, self-efficacy, and self-esteem and has been proposed as a determinant of health status. Thus, currently, the rehabilitation process tends to focus on improving the participation level among patients (6). When a person with MS starts a rehabilitation process, it is essential to identify the needs, based not only on the symptoms but also on the difficulties encountered in daily life. Standardized, valid, and reliable tools are essential to effective evaluation. Internationally, there are several evaluation scales capable of achieving these objectives (7). Compared to these scales, however, it was decided to use the Health Assessment Questionnaire (HAQ) because it has been translated into most languages and it has been classified as the best predictor of mortality, work disability, joint replacement, and medical costs. The US Food and Drug Administration accepts it as a measure for the evaluation of the prevention of disability (8). Furthermore, it is used in most clinical trials and observational outcome studies. To date, the HAQ questionnaire is available in English (9), Dutch (10), Swedish (11), Portuguese (Brasil) (12), French (13), Spanish (Mexico) (14), Spanish (Spain) (15), Italian (16), German (17), Arabic (Kuwait) (18), Korean (19), Chinese (Singapore) (20), Danish (21), Slovak (22), Indian (23), Arabic (Egypt) (24), Spanish (Argentine) (25), Estonian (26), Greek (27), Thai (28), Turkish (29), Bengali (30), Nepali (31), Malay (32), Persian (33), and Japanese (34).

The psychometric properties of the HAQ have never been studied in a population of people with neurological disease. The validation of this measurement tool for people with MS allows the comparison of studies that analyze the same treatment in different diseases. Moreover, using the same assessment tool can define which treatment is more effective than others. This study aims to evaluate the psychometric properties of HAQ in a population of individuals with MS and compare the results with their QOL.

## Methods

This study was conducted by a research group from “Sapienza” the University of Rome (35–44).

This cross-sectional study was performed in line with Consensus-based Standards for the selection of health Measurement Instruments (COSMIN); refer to the COSMIN checklist to examine the psychometric properties of the HAQ (45).

### Participants and Recruitment

A survey was conducted on a cohort of consecutive patients about the neurologic outpatient clinic at the Policlinic Umberto I in Rome between February and October 2020. There were no inclusion or exclusion criteria for participants except that they should be diagnosed with MS, per the “McDonald's” clinical diagnostic criteria for MS and a score with a range of 1–8 (46). Individuals in the MS Center of the clinic were verbally informed about the study's methods and purposes by their neurologist. All participants were informed about the study, and their interest in taking part was recorded; those who subsequently entered the study gave their written consent before inclusion (47, 48).

### Assessment Tools

1. The Expanded Disability Status Scale (EDSS) is the most commonly used scale in patients with MS. The EDSS is a very effective method of reflecting disability. The EDSS, with a scoring system between 0 and 10, reveals the patient's morbidity. Zero points are normal neurological examinations. Ten points show the MS-related death cases. Patients with an EDSS score up to 5 are fully ambulatory patients. Up to this point, the main determinant of EDSS are functional systems (FS), the ambulation status is the main determinant in the degree of disability after 5 (49);
2. The HAQ consists of 20 questions relating to the ability to carry out common daily life activities, which are divided into eight sections: dressing, arising, eating, walking, hygiene, reach, grip, and activities. There are four possible responses for each question, with the degree of difficulty that the requested action involves: 0 = without any difficulty; 1 = with some difficulty; 2 = with much difficulty; 3 = unable to do. For each category, the highest score is considered and the sum of the scores (from 0 to 24) divided by 8 represents the final HAQ score, which can vary from a minimum of 0 to a maximum of 3. The higher the score, the greater the disability; The time of administration ranges between 10 and 20 min (9);
3. The Short Form (36) Health Survey (SF-36) consists of eight scaled scores (vitality, physical functioning, bodily pain, general health perceptions, physical role functioning, emotional role functioning, social role functioning, and mental health). Each of those consists of 1–10 questions. Also, a single-question assessment on the change in health conditions is not used for scoring in any of the eight scales. Synthetic indices that globally describe the state of physical and mental health were obtained from the aggregation of the different subscales. The questions and subscales of SF-36 are organized so that the higher the score, the better the health of the subject. Standardized mathematical procedures establish the algorithms relating to the scoring phases (50).

### Data Collection

At the beginning of the study, the patients' data were collected (name and surname, date of birth, education, marital status, profession, city of residence, type of house, presence of architectural barriers, people living in the house, caregiver, if in possession of a mobility aid, years of duration of the illness, and availability for any future studies). The questionnaire choices were based on the study objectives. The EDSS, administered by the neurologist, was used to define the progression of the disease, SF-36, to evaluate the health state of patients, and HAQ to evaluate the limitations in the QOL due to disease.

Two occupational therapists administered the questionnaire. The patients were interviewed separately at a neurological clinic. The questionnaires are self-administered by the patient if he could, or by the caregiver if the patient was not cognitively fit.

### Data Analysis

All the data were coded, inputted, and analyzed into Microsoft Exce (One Microsoft Way, Redmond, Washington, USA). The data were analyzed with descriptive statistics measures. The reliability of the test was assessed by measuring the value of Cronbach's alpha for internal consistency. As recommended, the acceptable alpha coefficient was set to at least 0.70 (51). The correlations between the instruments were assessed using the Pearson correlation coefficient. The Pearson's correlation coefficient was interpreted as follows: 0 indicated no linear relationship; +1/−1 indicated a perfect linear relationship (positive/negative); a value between 0 and 0.3 indicated a weak linear relationship; values from 0.3 to 0.7 indicated a moderate linear relationship; values between 0.7 and 1 indicated a strong linear relationship (52). Any *p*-values ≤0.05 were considered statistically significant. All statistical analyses were performed using IBM-SPSS version 23.00 [International Business Machines Corporation (IBM), Armonk, New York, USA].

## Results

Table 1 presents the demographic and clinical characteristics of the study subjects. Of the 34 patients, 17 are women (50%) and 17 are men (50%), with a mean age of 49 years. A total of 4 patients (11.8%) are in EDSS grade 0, 2 (5.8%) in grades 1 and 1.5, 7 (20.5%) in grades 2 and 2.5, 2 (5.9%) in grade 3, 4 (11.7%) in grades 4 and 4.5, 3 (8.8%) in grade 5.5, 10 (29.4%) in grades 6 and 6.5, 1 (2.9%) in grade 7, and 1 (2.9%) in grade 8. Approximately 79% of the patients are diagnosed with relapsing-remitting MS (RRMS), and 21% are diagnosed with MS Secondarily Progressive (SMSP). Furthermore, 19 patients (55.9%) out of 34 reported being autonomous in the activities of daily life (ADL), and, therefore, they do not need a caregiver.

**Table 1:** Demographic and clinical characteristics of the participating population.

	**Mean (standard deviation)**
Age	49 (9)
EDSS	4 (2.5)
	Frequency (%)
**Diagnosis**
Relapsing remittent	27 (79.4)
Secondary progressive multiple scale	7 (20.6)
**Gender**
Women	17 (50.0)
**Education**
High school	19 (25.9)
Gratuated	7 (20.5)
Middle school	8 (23.6)
**Marital status**
Single	11 (32.3)
Married	16 (47.1)
Unmarried	5 (14.7)
Widowed	2 (5.9)
**Employment status**
Unemployed	7 (20.6)
Employed	17 (50.0)
Freelance professional	7 (20.6)
Retired	2 (5.9)
Student	1 (2.9)
**Caregiver**
Nobody	19 (55.9)
Formal	2 (5.9)
Informal	13 (38.2)

Table 2 shows the mean results and standard deviation of the HAQ score for each sub-category (dressing, arising, eating, walking, hygiene, reach, grip, and activity), highlighting the interrelationships. From these, a total Cronbach's alpha of 0.94 emerged.

**Table 2:** Internal consistency of the Health Assessment Questionnaire (HAQ).

	**Mean**	**Cronbach's alpha**
	**(standard deviation)**	**if item Deleted**
Dressing	0.88 (1.04)	0.931
Arising	1.12 (1.15)	0.925
Eating	0.94 (1.15)	0.936
Walking	1.24 (1.05)	0.932
Hygiene	1.18 (1.14)	0.930
Reach	1.06 (1.18)	0.932
Grip	0.41 (0.86)	0.946
Activity	1.41 (1.21)	0.929
Total alpha 0.94

Table 3 shows the correlation between the dimensions of SF-36, the sub-categories of the HAQ, and its total according to the Pearson Correlation Coefficient. To evaluate a possible linearity relationship between SF-36 and HAQ, the Pearson Correlation Coefficient was used, from which significant correlations (*p* < 0.01) emerged. As shown in Table 3, it was found that the eight sub-categories of the HAQ are inversely proportional to the dimension of physical functioning and activity limitations due to the physical health of SF-36. It also emerged that HAQ sub-categories are inversely proportional to the dimension of the limitations of activities due to emotional problems and energy/fatigue, especially in the grip, where there is a significant correlation at the 0.01 level. All items of the HAQ are inversely proportional to emotional wellbeing, significantly in eating and grip. There is a significant correlation (*p* < 0.01) between social functioning and eating, the action of arising, grip, reach, and activity; therefore, social functioning decreases as the difficulty in eating increases. The categories of arising and activity turn out to be significantly inversely proportional to general health. As it is possible to find in the current literature, invisible MS symptoms negatively affected the patients' social lives. Programs should be designed to improve the work integration and daily activities of patients with MS (53).

**Table 3:** Correlations between Health Survey—Short Form 36 (SF36) and the HAQ.

	**Physical**	**Role limitations due**	**Role limitations due**	**Energy/**	**Emotional**	**Social**	**Pain**	**General**
	**functioning**	**to physical health**	**to emotional problems**	**fatigue**	**wellbeing**	**functioning**		**health**
Dressing	−0.765^**^	−0.474^*^	−0.087	−0.246	−0.215	−0.249	−0.023	−0.309
Arising	−0.784^**^	−0.453^*^	−0.199	−0.305	−0.330	−0.403^*^	−0.170	−0.388^*^
Eating	−0.750^**^	−0.679^**^	−0.314	−0.374	−0.384^*^	−0.396^*^	−0.279	−0.327
Walking	−0.793^**^	−0.377^*^	−0.048	−0.306	−0.105	−0.218	−0.055	−0.260
Hygiene	−0.854^**^	−0.567^**^	−0.117	−0.363	−0.174	−0.249	−0.087	−0.343
Reach	−0.689^**^	−0.551^**^	−0.305	−0.286	−0.235	−0.400^*^	−0.207	−0.221
Grip	−0.421^*^	−0.465^*^	−0.445^*^	−0.417^*^	−0.469^*^	−0.494^**^	−0.298	−0.235
Activity	−0.840^**^	−0.600^**^	−0.267	−0.371	−0.287	−0.445^*^	−0.193	−0.377^*^

* *Correlation is significant at the 0.05 level (2-tailed)*.

** *Correlation is significant at the 0.01 level (2-tailed)*.

Table 4 shows the cross-cultural correlations between the clinical and demographic characteristics of the study participants, the sub-categories of the HAQ, and their total scores.

**Table 4:** Correlations between the clinical and demographic characteristics of the population and items of HAQ.

	**Dressing**	**Arising**	**Eating**	**Walking**	**Hygiene**	**Reach**	**Grip**	**Activity**	**HAQ total**
Type of MS	0.509^**^	0.568^**^	0.473^**^	0.281	0.388^*^	0.585^**^	0.280	0.486^**^	0.517^**^
Years from diagnosis	0.058	0.048	0.180	−0.073	−0.005	0.049	−0.220	0.056	0.090
EDSS	0.678^**^	0.802^**^	0.590^**^	0.798^**^	0.750^**^	0.560^**^	0.292	0.834^**^	0.829^**^
Age	0.333	0.194	0.180	0.078	0.384^*^	0.027	−0.104	0.344^*^	0.259
Gender	−0.129	−0.044	−0.058	−0.175	−0.044	−0.179	0.024	0.006	−0.102

* *Correlation is significant at the 0.05 level (2-tailed)*.

** *Correlation is significant at the 0.01 level (2-tailed)*.

## Discussion

This study evaluated the psychometric properties of HAQ in a population of individuals with MS and has been shown that these properties are satisfactory, with good reliability and validity, in patients with MS.

In the study, from the mean of the HAQ sub-categories' intercorrelations, it emerged that the total Cronbach's alpha is equal to 0.94 (Table 2). These data are in line with other studies, for example, the HAQ showed an alpha value of 0.90 in the Spanish (Mexican) version (54) and 0.98 in the Chinese version (55). Therefore, it has excellent internal consistency and good consistency between the items in the questionnaire. The Italian version of SF-36 was used as a gold standard to assess construct validity. The construct validity analysis showed a linear correlation between activity limitation and quality of life; this correlation was higher for physical functioning. A recent study of the Chinese version showed a strong correlation between HAQ in a patient with rheumatoid arthritis with SF-12, also for the mental health component (55).

Finally, a cross-cultural analysis was made with the HAQ, from which, as reported in Table 3, it emerged that according to the type of diagnosis (relapsing remittent or progressive), there is a significant direct proportionality with difficulty in all sub-categories of the HAQ and the total score of the HAQ, except for walking and grip. Furthermore, it is shown that the degree of EDSS of the subjects has a significant direct proportionality with all the items of the HAQ, except for grip; therefore, the higher the EDSS stage, the greater the difficulties in the aforementioned activities. This means that people with progressive MS and with higher levels of EDSS have a greater impact on ADLs. The last significant correlation occurs between the patient's age, hygiene, and activity in a directly proportional way.

### Limitations of the Study

The study's limitations are mainly due to the historical period in which it was conducted during the pandemic due to the severe acute respiratory syndrome coronavirus 2 (SARS-CoV-2) infection. For this reason, there was no possibility of administering the questionnaires to the population. Consequently, the number of participants is not very large. Future studies should include larger samples of patients with MS to overcome this limitation.

### Conclusions

In conclusion, with these psychometric properties, HAQ appears to be a valid tool to evaluate the ability to carry out in a population with MS. It could provide the healthcare and rehabilitation sector with an additional tool for assessing patients' disabilities with MS. The validation of this measurement tool for people with MS allows comparing outcomes of various studies. It is useful for testing the effectiveness of a treatment in various diseases, and using the same assessment tool can define which treatment is more effective than others.

## Data Availability Statement

The original contributions presented in the study are included in the article/supplementary material, further inquiries can be directed to the corresponding author.

## Ethics Statement

Ethical review and approval was not required for the study on human participants in accordance with the local legislation and institutional requirements. The patients/participants provided their written informed consent to participate in this study.

## Author Contributions

AB, GG, AC, and GF contributed to conception and design of the study. LC and CC organized the database. GG performed the statistical analysis. AB wrote the first draft of the manuscript. MTo, MTa, VB, and SC wrote sections of the manuscript. All authors contributed to manuscript revision, read, and approved the submitted version.

## Conflict of Interest

The authors declare that the research was conducted in the absence of any commercial or financial relationships that could be construed as a potential conflict of interest.

## Publisher's Note

All claims expressed in this article are solely those of the authors and do not necessarily represent those of their affiliated organizations, or those of the publisher, the editors and the reviewers. Any product that may be evaluated in this article, or claim that may be made by its manufacturer, is not guaranteed or endorsed by the publisher.
